# p53 Immunohistochemistry Expression in Epithelial Ovarian Tumors

**DOI:** 10.7759/cureus.107496

**Published:** 2026-04-21

**Authors:** Sushma Madini, T.N. Suresh, Abhay K Kattepur

**Affiliations:** 1 Pathology, Sri Devaraj Urs Medical College, Kolar, IND; 2 Pathology and Laboratory Medicine, Sri Devaraj Urs Medical College, Kolar, IND; 3 Surgical Oncology, Sri Devaraj Urs Medical College, Kolar, IND

**Keywords:** epithelial ovarian tumors, immunohistochemistry, ovarian carcinoma, p53, prognosis, tumour grade

## Abstract

Introduction

Epithelial ovarian carcinoma usually has TP53 expression. The TP53 gene is the most frequently mutated gene in ovarian cancer and therapies targeting these mutations may improve treatment outcomes, but there are only few studies on p53 immunohistochemistry expression in ovarian tumors in India, leading to this study.

Objective

To study the expression of p53 mutation in epithelial ovarian carcinoma and to find its association with histological type, grade, and TNM staging.

Methodology

This is a laboratory-based explorative study. Expression of p53 was examined in 62 cases of epithelial ovarian carcinoma. Key parameters, including age, site of distribution, histological variant, tumor grade, and TNM stage, were analyzed for correlations with p53 expression using the chi-square test.

Results

Out of 62 cases studied, serous cystadenocarcinoma was the largest group, with 53 cases (85.5%), followed by mucinous cystadenocarcinoma with five cases (8%) and sero-mucinous cystadenocarcinoma with four cases (6.4%). 77.4% of serous cystadenocarcinoma cases (41/53) and all four cases of sero-mucinous cystadenocarcinoma (100%) showed 5+ staining, while none of the mucinous cystadenocarcinoma cases exhibited 5+ expression. p53 expression significantly correlated with histological variant of tumor (*p* = 0.003). No association was found between the expression of p53 with TNM staging and tumor grade.

Conclusion

High p53 expression was seen in 72.5% (45 out of 62) of ovarian carcinoma cases. High p53 expression is strongly associated with serous cystadenocarcinoma and sero-mucinous cystadenocarcinoma as compared to mucinous cystadenocarcinoma.

## Introduction

Ovarian cancer (OC) is the eighth most prevalent incident cancer in women and the eighth leading cause of cancer-related deaths worldwide [1]. Carcinoma of ovary is the second most common gynaecological malignancy [2]. GLOBOCAN 2020 statistics show that OC is responsible for 2.1% of all site deaths and 1.6% of new cases [3]. Globally, ovarian cancer affects 238,719 women and accounts for over 150,000 deaths annually, largely attributed to late-stage diagnosis at presentation [4]. Epithelial ovarian cancer accounts for approximately 90% of all ovarian cancer cases [5]. Thirty percent to 80% of epithelial ovarian tumors have been found to have a mutation or deletion of the p53 tumor suppressor gene [6]. p53 controls various cellular processes, including apoptosis, cell cycle regulation, DNA repair and cell differentiation, by regulating key gene expression [7]. The most often mutated gene in human malignancies is p53 and the majority of epithelial ovarian tumors exhibit loss of p53 protein [8]. Recurrence occurs in 70% of cases with advanced disease within three years [9].

Maintenance therapy with olaparib (for BRCA mutation-positive) or bevacizumab (for others) delays recurrence but doesn’t improve overall survival. Both treatments have significant side effects [10]. A promising alternative is targeting p53 mutations, with PRIMA-1MET (APR-246) under clinical trials to restore normal p53 function [5]. There are only a few studies done on expression of p53 in ovarian tumors in India, leading to this study.

## Materials and methods

Methodology

It is a retrospective cross-sectional study of laboratory data review done on 62 cases of epithelial ovarian tumor that were received in the Department of Pathology, Sri Devaraj Urs Medical College, Tamaka, Kolar, between January 2016 and May 2024.

Inclusion criteria

All histological diagnoses of epithelial ovarian carcinoma specimens admitted to and resected in R L Jalappa Hospital and Research Institute affiliated to Sri Devaraj URS Academy of Higher Education were included.

Exclusion criteria

Patients who underwent chemotherapy for other cancer over the past five years were excluded.

Method of collecting data

Samples of the paraffin blocks and data from the medical records meeting the exclusion and inclusion criteria from the period of January 2016 to May 2024 as mentioned above were collected and analysed. The preserved samples were re-examined under the microscope and data compiled.

Statistical tests

Chi-square test was used as a test of significance.

Statistical test software

Data was collected with the help of a proforma, tabulated and entered into Excel (Microsoft, Redmond, WA, USA), then analyzed with SPSS 22 version software (IBM Corp., Armonk, NY, USA).

The present study was conducted to assess the expression of p53 mutation in epithelial ovarian carcinoma and determine its correlation with histological variant, grade, and TNM stage. Tissue sections were preserved and embedded with paraffin. p53 immunohistochemical stain was done in addition to hematoxylin and eosin staining (clone DO-7, Diagnostic Biosystems, Pleasanton, CA, USA) of primary mouse monoclonal antibody; 30 min with 1:200 dilution was done.

In this study, the immunohistochemical p53 was scored as follows [9]: Score 0 (negative/occasional positive cells); Score 1+ (10% cells positive); Score 2+ (10%-25% cells positive); Score 3+ (26%-50% cells positive); Score 4+ (51%-75% cells positive); Score 5+ (75% cells positive).

p53 staining was semi-quantitatively analyzed based on the nucleus's brown staining results.

## Results

Out of a total of 62 cases of epithelial ovarian tumors, 52 cases were serous carcinoma, five cases were mucinous carcinoma, and four cases were sero-mucinous carcinoma (Table 1). The maximum number of patients was in the 40 to 49 age group. In the present study, out of 62 cases, 28 and 16 cases were right and left ovary, respectively, whereas 18 cases were bilateral. It was observed that high-grade tumors were 52 cases and low-grade tumors were 10 cases. In T staging, the maximum number of cases, 35 (56.4%), were seen in T3. Only two cases showed lymph node metastasis (3.2%). Staging of the tumor was done according to International Federation of Gynecology and Obstetrics (FIGO), in which the majority of cases, 34 (54.8%), were seen in stage III (Table 1).

**Table 1:** Showing clinicopathological data of the studied cases (62 cases). FIGO: International Federation of Gynecology and Obstetrics

Clinicopathological data	Total cases (n=62)	% of cases
AGE OF DISTRIBUTION		
20-29	6	9.6%
30-39	9	14.5%
40-49	19	30.6%
50-59	8	12.9%
60-69	16	25.8%
70-79	4	6.4%
HISTOLOGICAL TYPE OF TUMOR		
Serous	53	85.4%
Mucinous	5	8.1%
Sero-mucinous	4	6.4%
TUMOR GRADE		
High Grade	52	83.8%
Low Grade	10	16.1%
T STAGING		
T1	27	43.5%
T2	0	0%
T3	35	56.4%
N STAGING		
N x	27	43.5%
N0	33	53.2%
N1	2	3.2%
FIGO STAGING		
I	27	43.5%
II	0	0%
III	34	54.8%
IV	1	1.6%

**Figure 1:**
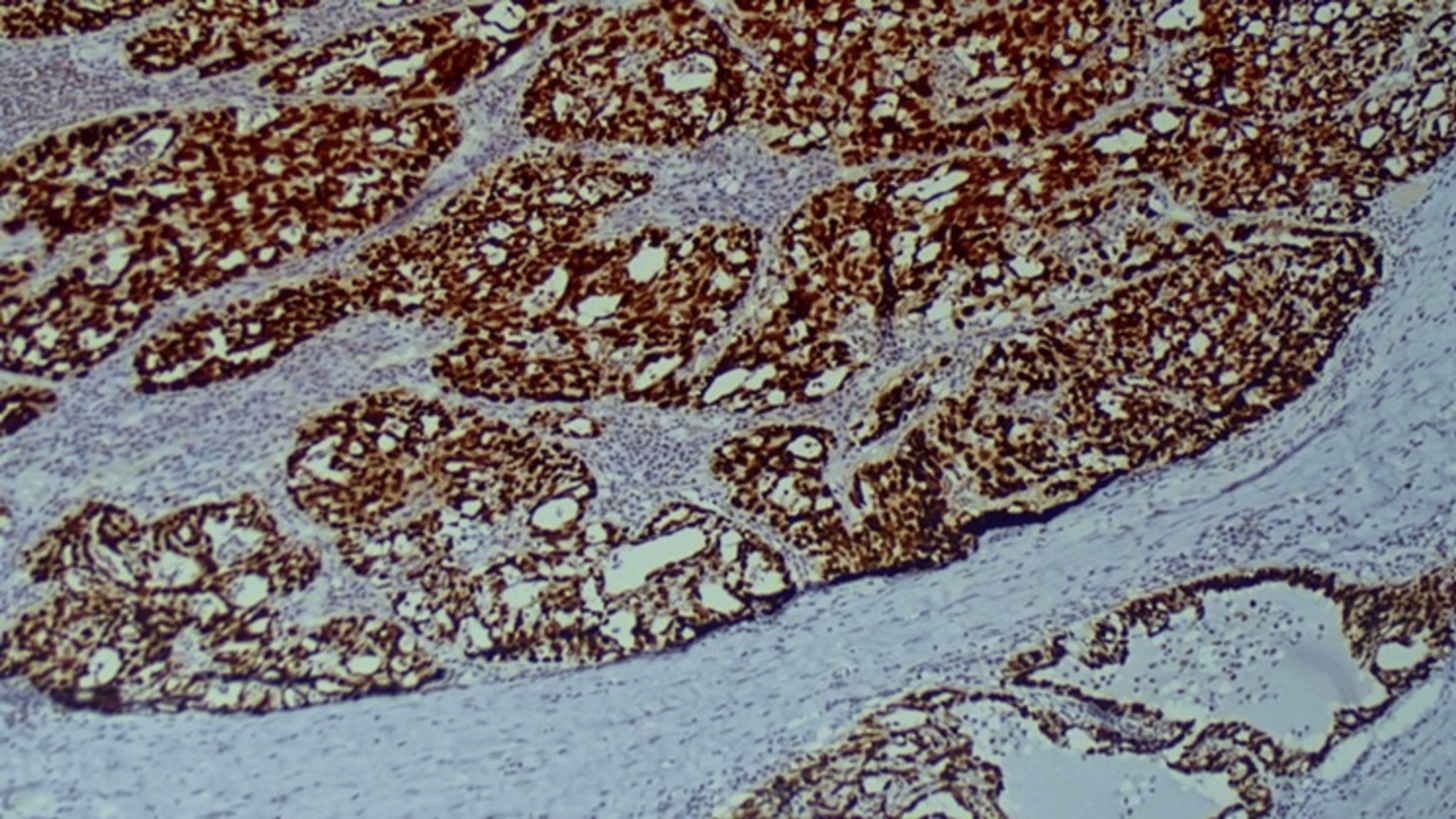
p53 Showing 5+ Scoring in Serous Carcinoma (IHC, 100x) IHC: Immunohistochemistry

77.4% of serous cystadenocarcinoma cases (41/53) and all four cases of sero-mucinous carcinoma (100%) showed 5+ staining (Figure 1), while none of the mucinous cystadenocarcinoma cases exhibited 5+ expression. p53 expression shows significant correlation with histological variant of carcinoma (p=0.003) (Table 2).

**Table 2:** Relation between p53 expression with clinicopathological data Pearson chi-square test was done to determine the P-value FIGO: International Federation of Gynecology and Obstetrics, IHC: Immunohistochemistry

Histological type of tumor	Total Cases (n=62)	p53 IHC EXPRESSION						P value	Pearson Chi-Square test
		0	1+	2+	3+	4+	5+		
Serous	53	0	2(3.7%)	1(1.8%)	3(5.6%)	6(11.3%)	41(77.4%)	0.003	Value = 23.669; df = 8; Asymp.Sig(2-sided) =0.003
Mucinous	5	0	0	2(40%)	1(20%)	2(40%)	0		
Seromucinous	4	0	0	0	0	0	4(100%)		
TUMOR GRADE									
HIGH GRADE	52	0	1(1.9%)	2(3.8%)	3(5.7%)	6(11.5%)	40(76.9%)	0.421	Value =3.888; df = 4; Asymp.Sig (2-sided) = 0.421
LOW GRADE	10	0	1(10%)	1(10%)	1(10%)	2(20%)	5(50%)		
T STAGING									
T1	27	0	0	1(3.7%)	2(7.4%)	2(7.4%)	22(81.5%)	0.496	Value = 3.380 df = 4 Asymp.Sig (2-sided) = 0.496
T2	0	0	0	0	0	0	0		
T3	35	0	2(5.7%)	2(5.7%)	2(5.7%)	6(17.1%)	23(65.7%)		
FIGO STAGING									
I	27	0	0	1(3.7%)	2(7.4%)	2(7.4%)	22(81.5%)	0.287	Value = 9.695 df = 8 Asymp.Sig (2-sided) = 0.287
II	0	0	0	0	0	0	0		
III	34	0	2(5.8%)	2(5.8%)	2(5.8%)	5(14.7%)	23(67.6%)		
IV	1	0	0	0	0	1(100%)	0		

5+ staining was observed in 76.9% of high-grade ovarian tumors and 50% of low-grade tumors. There is no statistically significant correlation between the grade of tumor and the expression of p53.

Expression of p53 with 5+ staining was observed in 81.5% (22 cases) of T1 stage and 65.7% (23 cases) of T2 stage. However, no statistically significant correlation between T staging and expression of p53 (p = 0.496) was seen. There were only two cases showing nodal metastasis.

Expression of p53 with 5+ staining was observed in 22 cases (81.5%) of Stage I and 23 cases (67.6%) of Stage III. However, no statistically significant correlation between FIGO staging and expression of p53 (p = 0.287) was seen (Table 2).

## Discussion

Ovarian tumors are diverse in morphology. Recent WHO classification divides them into type 1 and type 2 based on their biology and behavior [11]. Type 1 tumors, such as serous carcinoma of low-grade type, account for 10% of ovarian cancer deaths and are less aggressive, often diagnosed at earlier stages. In contrast, type 2 tumors, including serous carcinoma of high-grade type are aggressive, diagnosed at advanced stages and responsible for 90% of ovarian cancer deaths. These tumors develop rapidly, frequently causing ascites [12,13].

The 40-to-49-year-old age group accounted for the majority of ovarian cancers in the present study, consistent with the findings of Singh et al. [6], where the patient age range was 21-90 years, with mean age of 47 years. This is likely due to ovarian tumors being most commonly diagnosed in postmenopausal women.

Association between p53 mutations or overexpression and ovarian carcinoma subtypes is still unclear [14]. In the present study, 77.4% of serous cystadenocarcinoma cases (41/53) and all four sero-mucinous cystadenocarcinoma cases (100%) showed strong 5+ staining, while no mucinous cystadenocarcinoma cases showed 5+ expression. This association between p53 expression and serous histological type was supported by similar findings (Table 3) in studies done by Elnashar et al. [7] and Singh et al. [6].

**Table 3:** Comparative analysis of p53 expression with a score 5+ in histological type of tumor with other studies.

	Sample Size	p53 expression	
		Serous cystadenocarcinoma	Mucinous cystadenocarcinoma
Present study (2024)	62 cases	77.4% (41/53)	0% (0/5)
Elnashar AT et al. (2022) [7]	52 cases	94.5% (35/37)	0% (0/12)
Singh A et al. (2021) [6]	76 cases	45.2% (N/A)	29.2% (N/A)

Higher FIGO staging indicates more aggressive tumor biology, making it a significant independent prognostic factor [15]. In the present study, 43.5% of cases were in FIGO Stages I & II (27 cases), compared to 56.5% in Stages III & IV (35 cases). However, no statistically significant correlation between FIGO staging and expression of p53 (p = 0.287) was seen, likely due to the small sample size.

These findings were also consistent with a study conducted by Choudhury et al. [8], which reported 23% of cases in stages I-II and 60% cases in stages III-IV, with no statistical significance (P = 0.08). Similarly, in a study by Hartmann et al. [16], 56 cases (19.7%) were in stages I/II, while 234 cases (80.2%) were in stages III/IV, also showing no statistically significant association between FIGO staging with expression of p53 (P = 0.13).

In the present study, 76.9% of cases showed high-grade type of tumor and 50% of cases showed low-grade type of tumor; no statistically significant correlation between expression of p53 and tumor grade was seen. This contrasts with the findings of Mishra et al. [9], where only serous carcinoma cases were analyzed, where 64% cases were tumors of high-grade type and 18% cases were tumors of low-grade type, potentially leading to significant expression of p53 and tumor grade. In a study conducted by Hartmann et al. [16], 234 cases (82.4%) were classified as high grade, while 50 cases (17.6%) were in low grade. Due to the large sample size, a statistically significant association was found between expression of p53 and grade of tumor (P = 0.003).

High expression of p53 was observed in 72.5% (45 out of 62) of ovarian carcinoma cases. This elevated expression of p53 was strongly associated with cystadenocarcinoma of serous type and sero-mucinous type, as compared to cystadenocarcinoma of mucinous type, where the expression was significantly lower. Histological type and expression of p53 was statistically significant (P = 0.003), indicating that certain types of ovarian carcinoma, particularly serous and sero-mucinous subtypes, are more likely to exhibit higher expression of p53.

However, no significant correlation was found between expression of p53 and other factors such as TNM staging or grade of tumor.

Limitations

This study is a retrospective study with non-randomized data with a small sample size.

## Conclusions

Expression of p53 is higher in high-grade types of tumors like carcinoma of the serous type and sero-mucinous type. Research with larger sample sizes and higher expression of p53 levels is important to confirm that p53 can serve as a biomarker to help guide prognosis and treatment for aggressive tumors like serous carcinoma and sero-mucinous carcinoma.
